# *Leishmania* infection in blood donors: A new challenge in leishmaniasis transmission?

**DOI:** 10.1371/journal.pone.0198199

**Published:** 2018-06-14

**Authors:** Adriana de Oliveira França, Mauricio Antonio Pompilio, Elenir Rose Jardim Cury Pontes, Márcia Pereira de Oliveira, Luiza Oliveira Ramos Pereira, Rosimar Baptista Lima, Hiro Goto, Maria Carmen Arroyo Sanchez, Mahyumi Fujimori, Manoel Sebastião da Costa Lima-Júnior, Maria de Fatima Cepa Matos, Maria Elizabeth Moraes Cavalheiros Dorval

**Affiliations:** 1 Laboratory of Clinical Parasitology, Graduate Program in Infectious and Parasitic Diseases, Universidade Federal de Mato Grosso do Sul, Campo Grande, Mato Grosso do Sul, Brazil; 2 Hélio Mandetta School of Medicine, Universidade Federal de Mato Grosso do Sul, Campo Grande, Mato Grosso do Sul, Brazil; 3 Graduate Program in Infectious and Parasitic Diseases, Universidade Federal de Mato Grosso do Sul, Campo Grande, Mato Grosso do Sul, Brazil; 4 Interdisciplinary Laboratory of Medical Research (LIPMed), Instituto Oswaldo Cruz, Rio de Janeiro, Rio de Janeiro, Brazil; 5 Laboratory of Seroepidemiology and Immunobiology, São Paulo Institute of Tropical Medicine, Universidade de São Paulo, São Paulo, São Paulo, Brazil; 6 Laboratory of Immunopathology and Molecular Biology, Fundação Oswaldo Cruz, Ageu Magalhães Research Center (CPqAM), Recife, Pernambuco, Brazil; 7 Laboratory of Molecular Biology and Cell Culture, Universidade Federal de Mato Grosso do Sul, Campo Grande, Mato Grosso do Sul, Brazil; Taibah University, SAUDI ARABIA

## Abstract

Transfusion-transmitted leishmaniasis has been a concern in regions endemic for the disease. Whether immediate or delayed, the risks posed by this mode of transmission call for careful assessment. The purpose of this study was to detect *Leishmania* infection in blood donors living in an endemic area and to investigate progression to the disease in these individuals. Immunofluorescent antibody test, enzyme-linked immunosorbent assay, leishmaniasis rapid test, and the polymerase chain reaction were applied to 430 donors in an initial evaluation. Of those donors with at least one positive test, 50 were reevaluated four years later by the same methods, as were 25 controls who had been negative on the same tests. In the first evaluation, *Leishmania* infection was detected in 41.4% (95% CI: 36.7–46.1) of donors (*n* = 430). None of the 75 reevaluated individuals had developed the disease, but retesting revealed positivity in at least one test in 36.0% (95% CI: 25.1–46.9) of donors. Of the 50 initially testing positive, 50% remained so on retesting. Of the 25 initially negative controls, two tested positive in the subsequent evaluation. The severity of the parasitosis and the risk of transfusion transmission warrant investigation of the potential inclusion of methods for *Leishmania* detection into blood banks for effective screening of infected donors.

## Introduction

The ratio of asymptomatic *Leishmania* infections *versus* clinical cases is 13:1 in Iran [1] and 50:1 in Spain [2]. In Brazil, this ratio ranges from 8:1 to 18:1 [3], with evidence that one in every six infected individuals develops visceral leishmaniasis (VL) [4].

*Leishmania* transmission during blood transfusion has been a major concern in endemic areas. Although carriers may not exhibit clinical evidence of the disease, the parasite can become active and multiply in the mononuclear phagocytic system in response to factors such as patient immunological and nutritional status [5,6].

Infectivity among blood donors and the risk that latent infection becomes manifest appear to be self-perpetuating traits [7]. Asymptomatic infection in seemingly healthy donors promotes transfusion transmission [8] if parasite load is sufficiently high and amastigotes survive blood processing and storage until transfusion time [5]. This is a concern particularly among recipients exhibiting unfavorable immunological conditions, irrespective of exposure time [9].

The purpose of this study was to employ serological testing and identification of parasite DNA to detect cases of *Leishmania* infection in blood donors, thus providing support for discussions on the inclusion of laboratory methods for donor screening and selection in blood banks located in endemic regions. To this end, donors who initially tested positive on at least one of four diagnostic techniques were reevaluated after four years for observation of progression to the disease.

## Materials and methods

### Study population

The investigation was conducted at the José Scaff Hematology and Hemotherapy Center of Mato Grosso do Sul (Hemosul), in Campo Grande, the capital city of Mato Grosso do Sul state, in Midwest Brazil.

### Study design

The study comprised two evaluations. In the first, conducted in 2011, the indirect fluorescent antibody test (IFAT), enzyme-linked immunosorbent assay (ELISA rk39), rK39 rapid test, and polymerase chain reaction (kDNA-PCR) were applied to 430 donors. For the calculation of the sample, the average of 100 donors/day, five days per week was considered. For an approximate population of 14,000 individuals in the seven-month period, the sample size was recommended according to http://www.raosoft.com/samplesize.html, with a margin of error of 5% and confidence level equal to 95%. Systematic sampling was employed.

Subjects considered clinically fit for blood donation (Hemosul criteria) and having no signs, symptoms, or history of leishmaniasis were enrolled. Individuals seropositive for *Trypanosoma cruzi* were excluded. Infection with *Leishmania* sp., defined as positivity on at least one test, was detected in 178 subjects (41.4%).

Four years later, these 178 donors were invited for clinical evaluation by an infectologist and collection of a new blood sample at the Hospital Dia (a division of the teaching hospital of the Universidade Federal de Mato Grosso do Sul—UFMS). Changes of address or unavailability for the appointment, however, greatly reduced attendance to 75 individuals—namely, 50 donors who had tested positive on PCR, IFAT, or on both tests four years earlier, plus 25 donors testing negative on all exams (control group).

### Blood collection

A 10 mL blood sample was collected from each individual (7 mL for serum separation and 3 mL, preserved in EDTA, for DNA isolation). The samples were centrifuged and serum stored at –20 °C. DNA aliquots were maintained at –70 °C. The serological and molecular tests were performed in the UFMS Clinical Immunology Laboratory and the UFMS Molecular Biology and Cell Culture Laboratory, with support from the Oswaldo Cruz Institute Interdisciplinary Laboratory of Medical Research (LIPMed-Fiocruz, Rio de Janeiro) and the Seroepidemiology and Immunobiology Laboratory of the São Paulo Institute of Tropical Medicine (IMT-USP, São Paulo). All samples were subjected to IFAT, rK39 ELISA, rK39 rapid test, and kDNA-PCR.

Four years later, 75 blood samples were drawn and all the exams were repeated to assess the dynamics of infection.

#### Indirect fluorescent antibody test (IFAT)

Sera were subjected to IFAT [10] using a kit from the Instituto Biomanguinhos (Oswaldo Cruz Institute, Rio de Janeiro), following manufacturer’s instructions. Samples with IFAT titers of 1:80 or higher were considered positive. *Leishmania major*–like antigens obtained from cell culture were employed. The assay involved titration of the conjugate and inclusion of a negative and a positive control on each slide. The slides were read by two independent laboratory technicians [10].

#### Enzyme-linked immunosorbent assay (rK39 ELISA)

*Leishmania infantum* rK39 antigen was employed [11], using a modified technique [12]. Briefly, Costar High Binding 3690 polystyrene plates (Corning, Corning, NY, USA) were sensitized with 50 μL per well of 0.5 μg/mL of K39 recombinant antigen and blocked with 5% skimmed milk. Duplicate serum samples diluted 1:100 and anti-human IgG—peroxidase conjugate (A-0170, Sigma-Aldrich, St. Louis, USA) diluted 1:30 000 were incubated at 37 °C for 30 min. For color development, the samples were incubated in the dark with tetramethylbenzidine/H_2_O_2_ (Novex-Life Technologies, Carlsbad, CA, USA) (50 μL per well) at ambient temperature for 7 min. The reaction was quenched by adding 2 N H_2_SO_4_ (Merck KGaA). Absorbance was read at 450 nm on a Multiskan GO device (Thermo Scientific, Vantaa, Finland).

Cutoff was calculated based on a receiver operating characteristic (ROC) curve constructed from the absorbance values of 110 serum samples from patients with symptomatic, parasitologically confirmed VL who resided in an endemic area and 110 serum samples from São Paulo—based controls [13,14]. rK39 ELISA exhibited 99.1% sensitivity (95% CI: 95.0–100.0) and 100.0% specificity (95% CI: 99.1–100.0), considering a 0.110 cutoff. A reactivity index (*RI*) was calculated for each sample as *RI* = (*sample absorbance*)/*cutoff*. Samples with *RI* ≥ 1 were considered positive.

#### Anti-*Leishmania* rK39 rapid test

The rapid test employed a Kalazar Detect kit (Inbios International, Seattle, WA, USA), according to manufacturer’s instructions. Briefly, 10 μL of serum were placed in the specific area on the test strip and three drops of chase buffer were added. Results, read at 15 min, were considered positive or negative depending on the presence or absence of a test line, respectively. All tests were read by two technicians.

#### Polymerase chain reaction (PCR)

DNA isolation from blood was performed using a Wizard Genomic DNA Purification kit (Promega), according to manufacturer’s instructions. A human β-actin gene was used as a control to verify DNA integrity and the presence of possible PCR inhibitors [15].

*Primers* HM1 (5'-CCG CCC CTA TTT TAC ACC AAC CCC-3'), HM2 (5'-GGG GAG GGG CGT TCT GCG AA-3'), and HM3 (5'-GGC CCA CTA TAT TAC ACC AAC CCC-3') were employed to amplify the 120-bp fragment of the conserved region of *Leishmania* kDNA minicircle [16].

kDNA-PCR was performed with a final 25 μL volume containing 1x Colorless GoTaq Flexi buffer (Promega, Madison, USA), 200 μM dNTPs (dATP, dCTP, dGTP, and dTTP; Promega), 1.5 mM MgCl_2_, a 1 μM concentration of each *primer*, roughly 150 ng of extracted DNA, and water to complete the reaction. In all reactions, 2 μL of *Leishmania* (*Viannia*) *braziliensis* genomic DNA (MHOM/BR/1975/M2903, LIPMed-Fiocruz, Rio de Janeiro) served as the positive control. The negative control was a sample containing the reagent mixture devoid of DNA. Cycling started at 95 °C for 2 min, followed by 35 cycles at 95 °C for 30 s, 54 °C for 30 s, 72 °C for 30 s, and a final extension at 72 °C for 7 min.

Amplification was verified by performing 2% agarose gel electrophoresis in 0.5x TBE buffer, followed by gel staining with GelRed (1:500).

### Statistical analysis

BioEstat v. 5.3 software (Sociedade Mamirauá, Belém, Brazil) was employed to evaluate agreement between *Leishmania* detection tests, based on the kappa statistic (κ) at a 5% significance level, as follows: κ < 0.00, poor; κ = 0.00–0.20, slight; κ = 0.21–0.40, fair; κ = 0.41–0.60, moderate; κ = 0.61–0.80, substantial; κ = 0.81–1.00, almost perfect [17]. In the second evaluation, the clinical background and clinical status of participants were investigated by means of semistructured interviews, treatment history records, and general and specific physical examination.

### Ethical considerations

The study was approved by the Human Research Ethics Committee of the Universidade Federal de Mato Grosso do Sul (permit 0037.0.049.049–11). All subjects voluntarily signed a statement of informed consent for the collection of data and received their exam results, along with clarifications on clinical and epidemiological aspects of the infection.

## Results

Of the initial 430 blood donors, 70.2% were male and 29.8% female. Age ranged from 18 to 68 years, with a mean of 32 ± 10 years (SD). Of the 430 subjects, 131 (30.5%) were first- or second-time donors. The remaining 299 had been donors for 1 to 35 years, with a mean of 7 ± 6 years (SD). Of these, only 13.0% (39/299) did not donate blood on a regular basis.

In the first evaluation (*n* = 430), *Leishmania* sp. detection rates were as follows: 15.6% (95% CI: 12.2–19.0) on IFAT, 5.8% on rK39 ELISA (95% CI: 3.6–8.0), 12.1% on the rK39 rapid test (95% CI: 9.0–15.2), and 22.3% on PCR (95% CI: 18.4–26.3). Only one donor tested positive on all four methods.

Of the 96 donors who tested positive on PCR, 37 (38.5%) were also positive on IFAT. Of the 67 who were positive on IFAT, 37 (55.2%) were also positive on PCR (Table 1). Agreement between these techniques was fair (κ = 0.331, *p* < 0.001). Of the 67 donors positive on IFAT, seven (10.4%) were also positive on rK39 ELISA. Of the 25 positive on rK39 ELISA, seven (28.0%) were also positive on IFAT. Slight agreement was observed between these techniques (κ = 0.074, *p* = 0.039). No agreement was detected between the other tests.

**Table 1:** Distribution of blood donors in the initial evaluation, by test employed for *Leishmania* sp. detection (*n* = 430).

Test	*N*		κ	*p*
	Positive	Negative		
PCR				
IFAT				
Positive	37	30	0.331	<0.001
Negative	59	304		
Rapid test				
Positive	12	40	0.006	0.445
Negative	84	294		
rK39 ELISA				
Positive	7	18	0.026	0.241
Negative	89	316		
IFAT				
Rapid test				
Positive	8	44	-0.002	0.483
Negative	59	319		
ELISA-rK39				
Positive	7	18	0.074	0.039
Negative	60	345		
Rapid test				
ELISA-rK39				
Positive	1	24	-0.057	0.101
Negative	51	354		

**Fig 1:**
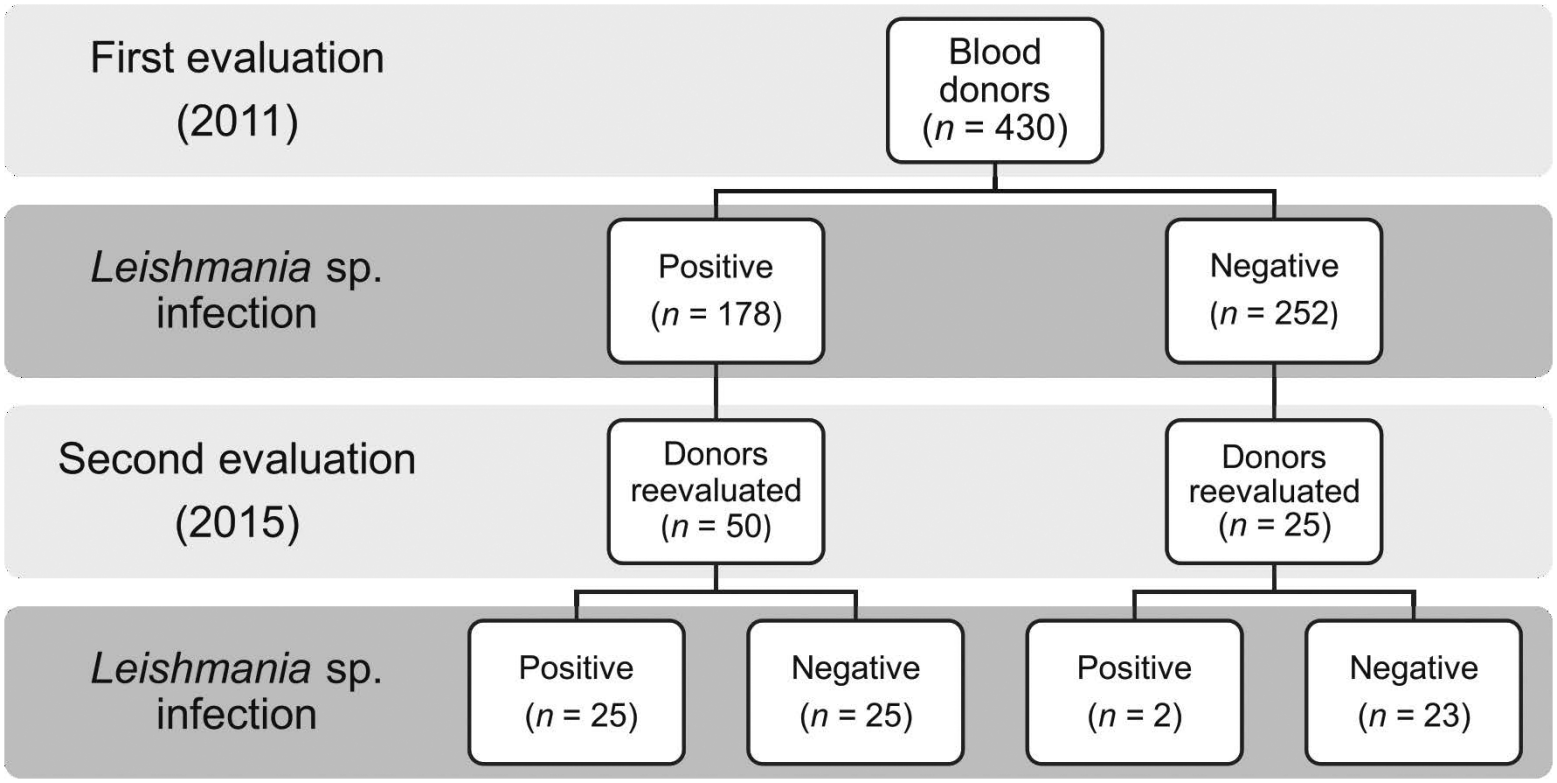
Distribution of blood donors, by positivity for *Leishmania* sp. on at least one test (IFAT, rK39 ELISA, rK39 rapid test, PCR).

In the initial evaluation, 41.4% of subjects (178/430; 95% CI: 36.7–46.1) were positive for at least one *Leishmania* test (Fig 1). Four years later, none had developed the disease, but infection was detected in 36.0% (27/75; 95% CI: 25.1–46.9). Of the 50 who initially tested positive in at least one test, 50.0% (95% CI: 36.6–63.4) retained this status. Of the 25 initially negative, two (8.0%) tested positive on the second evaluation.

Of the 50 subjects positive in the first evaluation, in at least one test, 16 (32%) were positive on IFAT, two (4%) on rk39 ELISA, and 14 (28%) on PCR, while all tested negative on the rK39 rapid test. Of the 25 individuals initially negative on all tests, two (8%) were positive on the IFAT test alone four years later.

Of those 14 positive on PCR in the second evaluation, two (14.3%) tested positive on rk39 ELISA, but a fair agreement was found between these techniques (κ = 0.213, *p* = 0.001). No agreement was detected between other tests (Table 2).

**Table 2:** Distribution of blood donors in the second evaluation, by test employed for *Leishmania* sp. detection (*n* = 75).

Test	*N*		κ	*p*
	Positive	Negative		
PCR				
IFAT				
Positive	5	13	0.130	0.128
Negative	9	48		
Rapid test				
Positive	-	-	-	-
Negative	14	61		
rK39 ELISA				
Positive	2	-	0.213	0.001
Negative	12	61		
IFAT				
Rapid test				
Positive	-	-	-	-
Negative	18	57		
rK39 ELISA				
Positive	1	1	0.055	0.191
Negative	17	56		
Rapid test				
rK39 ELISA				
Positive	-	2	-	-
Negative	-	73		

Comparison of both evaluations for each test (Table 3) revealed a fair agreement for rK39 ELISA (κ = 0.309, *p* = 0.002) and IFAT (κ = 0.302, *p* = 0.003), but slight agreement for PCR (κ = 0.171, *p* = 0.015). For presence of infection, defined by positivity on at least one of the four tests, a fair agreement was observed between first and second evaluations (κ = 0.341, *p* < 0.001).

**Table 3:** Distribution of blood donors for each test employed for *Leishmania* sp. detection in comparison of year of evaluation 2011 vs. 2015 (*n* = 75).

Test	2015		κ	*p*
	Positive	Negative		
2011	*N*	*N*		
rK39 ELISA				
Positive	1	3	0.309	0.002
Negative	1	70		
IFAT				
Positive	11	15	0.302	0.003
Negative	7	42		
PCR				
Positive	12	33	0.171	0.015
Negative	2	28		
Rapid test				
Positive	-	11	-	-
Negative	-	64		
*Leishmania* infection*				
Positive	25	25	0.341	<0.001
Negative	2	23		

*Defined as positivity on at least one test (IFAT, rK39 ELISA, rK39 rapid test, PCR).

The second evaluation revealed no subjects with a history of VL diagnosis or clinical treatment in the four-year study period investigated. No subjects developed visceromegaly or lymphadenopathy.

## Discussion

Among healthy populations, the seroprevalence of asymptomatic leishmaniasis ranges from <10.0% in regions where transmission rates are low or moderate [18], to >30.0% in areas with high transmission rates or among close contacts [19,20].

The first evaluation revealed infection in 41.4% of blood donors—a surprisingly high rate, not only for Brazil (Salvador: 5.4% of 700; Paraná: 11.4% of 176; Fortaleza: 17.1% of 431) [21–23], but also globally (France: 13.4% of 565; Spain: 3.1% of 1437; Nepal: 1% of 507) [24–26].

These disparate rates may be explained not only by regional variability in transmission dynamics, but also by differences in type and sensitivity of diagnostic methods [27] and the age range and specificities of the population investigated [20,28].

In the present study, four diagnostic methods were applied to all samples, whereas performing only molecular analysis of serologically positive samples can lead to underestimation of infection rates. In fact, prevalence and distribution of asymptomatic infection can serve as indicators of transmission, facilitating disease monitoring and control [29].

Presence of seropositive individuals in a population may indicate recent infection, followed by spontaneous cure [28,30], as seems to have occurred in the present sample, since none of the infected subjects developed the disease during the four-year study period investigated. However, longer exposure to the parasite may increase resistance against the disease, yielding positive tests in individuals with no history of VL [31]. In endemic areas, detection of antibodies can be interpreted as transient protection acquired from previous exposure, not necessarily indicating risk of disease progression [32]. As the receptors could not be investigated and the duration of the presence of these antibodies is not known, this possibility was not discussed.

Positivity on IFAT was defined as titers of 1:80 or higher—a criterion advocated by the Brazilian Ministry of Health [33] and adopted by a number of studies [20,31,32,34]—whereas adopting a 1:40 threshold [8,35] would have yielded a much higher prevalence rate.

In the present study, IFAT yielded higher positivity than other serological methods. Of the four tests employed for reevaluation, IFAT also yielded the highest rate of detection, despite the possibility of reinfection, with a consequent effect on anti-*Leishmania* antibody production. Differences in detection rates were also found for ELISA (25 positive cases, or 5.8%) and the rapid test (52, or 12.1%), both of which employed the rK39 antigen, which demonstrates that antigen choice can also influence antibody identification results [21]. Validated in Brazil and elsewhere [36,37], the rk39 rapid test has been used to detect asymptomatic and subclinical infections [29]. In the reevaluation, only two samples were positive on ELISA, while all were negative on the rapid test—a detection failure also observed in other studies [38,39].

The poor agreement observed among serological tests may stem from the fact that each detects a different stage of infection [32], with variable performance, given the diversity exhibited by the parasite and differences in antibody concentration, immune response, age range, and host nutritional status [40]. To overcome the limitations of serological detection and difficulties in parasite visualization, an association of methods has been recommended as an approach to enhance sensitivity and improve the detection of carriers [9,24,41].

Presence of amastigotes in peripheral blood of the donors was revealed by detection of *Leishmania* sp. DNA by PCR. Owing to its high specificity and sensitivity, the technique can detect low parasitic loads [42], even before clinical manifestation [43].

Amplification of *Leishmania* kDNA in serologically negative samples may indicate prior contact with the parasite, suggesting that kDNA presence, identified by PCR, is not always sufficient to elicit detectable humoral response [25,44–46]. On the other hand, adaptive immunity with antibody production, found in donors simultaneously positive on serology and negative on PCR, confirmed that *L*. *infantum* circulates intermittently, despite undetectable DNA [5,9,24].

No cases of progression to leishmaniasis were identified. Disease development, or lack thereof, may be related to exposure risk, geographic differences, or genetic susceptibility or resistance [46–48]. Although the mechanism of *Leishmania* survival in carriers remains unknown, the process is believed to dependent primarily on an equilibrium between host immune system and parasite virulence [24,41].

Although conversion rates can be significantly higher among individuals with positive results in more than one type of test [49], in the present study only one subject was positive on all tests in the first evaluation. Four years later, positivity in this donor was found on only two tests (ELISA and PCR), but no clinical manifestation was observed.

The high percentage of donors who were positive on both serology and PCR is a disquieting finding, compounded by their continued positive status four years later, irrespective of whether reinfection had occurred. Asymptomatic donors pose a silent threat to blood recipients—potential transmission has been illustrated by detection of free parasites in blood products following monocyte damage during fractionation [25]. Amastigote viability in blood under normal storage conditions [50] and experimental transmission of *Leishmania* [51,52] have both been confirmed.

The rising number of immunocompromised individuals is now a major contributor to the spread of infection, given their increased vulnerability to primary and reactivated infection with *Leishmania* [5,9]. In the present study it was not possible to obtain information on blood recipients, for the tracing and examination of them, since this process is totally confidential.

If better elucidated, asymptomatic cases should help in characterizing the epidemiology of leishmaniasis more accurately, serving as markers of potential progression to the disease, particularly in view of the frequency of seropositivity among the general population. Deeper knowledge of asymptomatic leishmaniasis can provide a fuller understanding of *Leishmania* transmission, dissemination, and survival in carriers, facilitating patient follow-up and ultimately reducing morbidity and mortality rates [34].

The implementation of routine screening methods in blood banks should improve the quality of donor selection, with immediate gains in the safety of blood products. For immunosuppressed recipients, inactivation techniques for a range of pathogens should be made mandatory [28], as deployed in the form of leucodepletion filters and other methods for *Leishmania* [53–56].

Special attention should be devoted to asymptomatic infection in blood donors recruited for simultaneous donation of bone marrow, given the potential presence of amastigotes in this type of tissue. Recent data on bone marrow recipients [38] suggest the advantages of employing PCR to identify higher risks of VL reactivation or protozoan transmission. Transmission by blood transfusion further complicates the clinical course of organ transplant, with possibly fatal outcomes [57].

The severity of this parasitosis and the risk of transfusion transmission, particularly in endemic areas, warrant investigation into the potential inclusion of methods for *Leishmania* detection into the routine of blood banks to ensure proper screening of donors. Although asymptomatic carriers are potential reservoirs, molecular studies are needed to evaluate the risks of parasite viability and development in the blood of donors and subsequent establishment of active disease in recipients.

## Supporting information

S1 Data available Tests. (XLS)

S2 Data available Tests and infection. (XLSX)
